# The *Xanthomonas* Ax21 protein is processed by the general secretory system and is secreted in association with outer membrane vesicles

**DOI:** 10.7717/peerj.242

**Published:** 2014-01-07

**Authors:** Ofir Bahar, Rory Pruitt, Dee Dee Luu, Benjamin Schwessinger, Arsalan Daudi, Furong Liu, Randy Ruan, Lisa Fontaine-Bodin, Ralf Koebnik, Pamela Ronald

**Affiliations:** 1 Department of Plant Pathology and the Genome Center, University of California, Davis, CA, USA; 2 UMR 186 IRD-Cirad-Université Montpellier 2 “Résistance des Plantes aux Bioaggresseurs”, Montpellier, France

**Keywords:** Xanthomonas, Rice, XA21, Outer membrane vesicles, Secretion, PAMPs, Plant immunity

## Abstract

Pattern recognition receptors (PRRs) play an important role in detecting invading pathogens and mounting a robust defense response to restrict infection. In rice, one of the best characterized PRRs is XA21, a leucine rich repeat receptor-like kinase that confers broad-spectrum resistance to multiple strains of the bacterial pathogen *Xanthomonas oryzae* pv. *oryzae* (*Xoo*). In 2009 we reported that an *Xoo* protein, called Ax21, is secreted by a type I-secretion system and that it serves to activate XA21-mediated immunity. This report has recently been retracted. Here we present data that corrects our previous model. We first show that Ax21 secretion does not depend on the predicted type I secretion system and that it is processed by the general secretion (Sec) system. We further show that Ax21 is an outer membrane protein, secreted in association with outer membrane vesicles. Finally, we provide data showing that *ax21* knockout strains do not overcome XA21-mediated immunity.

## Introduction

Pattern recognition receptors (PRRs) are proteins of critical importance to innate immunity in plants and animals. Upon recognition of conserved microbial signatures (also known as pathogen-associated molecular patterns, PAMPs), PRRs trigger immune responses that restrict infection (Schwessinger & Ronald, 2012). The rice PRR XA21 confers robust resistance to multiple strains of the rice pathogen *Xanthomonas oryzae* pv. *oryzae* (*Xoo*) (Song et al., 1995; Wang et al., 1996).

Over the last 10 years, several bacterial genes that are required for activation of XA21-mediated immunity (*rax* genes) have been identified (Shen et al., 2002; Burdman et al., 2004; Da Silva et al., 2004). These include three genes found in a single operon, *raxST*, *raxA*, and *raxB* (*raxSTAB*). *RaxST*encodes a tyrosine sulfotransferase (Da Silva et al., 2004; Han et al., 2012). RaxB is a predicted ATP-binding cassette (ABC) transporter that belongs to a distinct class of ABC transporters. This class of transporters contains an N-terminal C39 peptidase domain, characteristic of ABC transporters involved in secretion of peptides containing a double glycine- (GG-) leader peptide (Michiels et al., 2001; Da Silva et al., 2004). RaxA is a predicted membrane fusion protein that spans the periplasmic space (Da Silva et al., 2004). A fourth component, *raxC*, located outside the *raxSTAB* locus, is predicted to encode the outer membrane component of type I secretion systems (Holland, Schmitt & Young, 2005; Da Silva et al., 2004). *Xoo* strains mutated in *raxST*, *raxA*, *raxB* or *raxC* can evade XA21-mediated immunity to different degrees and form long disease lesions on rice expressing the XA21 receptor (Da Silva et al., 2004). Based on these observations, we hypothesized that the activator of XA21-mediated immunity is a RaxST-sulfated and RaxABC-secreted protein (Da Silva et al., 2004; Lee et al., 2006).

In 2009, we reported the identification of an *Xoo* protein (PXO_03968), that we named Ax21 (for Activator of XA21-mediated immunity) (Lee et al., 2009). Ax21 is a 198 aa-long protein that is conserved in several *Xanthomonas* species, *Xylella* and the human pathogen *Stenotrophomonas maltophilia*. Using silver staining and mass spectrometry analysis, we showed that *raxA* and *raxC* are required for Ax21 secretion. We also presented evidence that a mutation in *ax21* allowed the bacterium to evade XA21-mediated immunity. We further provided data indicating that a small, sulfated peptide, derived from the N-terminus of Ax21, called axY ^S^22, triggers XA21-mediated immunity. We have discovered errors in the study and have therefore retracted the paper (Lee et al., 2013). Details concerning the retraction are described in Lee et al. (2013), Han et al. (2013) and Ronald (2013).

Some of the retracted results have been reproduced in other laboratories (Shuguo et al., 2012; Wang et al., 2013). For example, Shuguo et al. (2012) showed that a sulfated, 36-aa peptide, derived from the N-terminus of Ax21, can induce various defense responses in XA21-expressing rice such as accumulation of reactive oxygen species, mitogen-activated protein kinase activation and *in planta* XA21-mediated immunity. Wang et al. (2013) showed that *ax21* deletion mutants in both *Xoo* (strain PXO99) and *X. oryzae* pv. *oryzicola* (*Xoc)* RS105 can evade XA21-mediated immunity. They further showed that Ax21 secretion in *Xoc* RS105 depends on RaxA and that supernatants from the *Xoc ax21* deletion mutant cannot induce XA21-mediated resistance, while supernatants from the wild type strain can.

Subsequent research from our lab, focused on the biological function of Ax21 in *Xanthomonas*, suggested that Ax21 serves as a cell–cell signaling molecule mediating motility, biofilm formation and virulence (Han et al., 2011). However, this report was also retracted (Ronald, 2013; Han et al., 2013). Here too, several labs have reproduced some of our results in other bacterial species (McCarthy, Dow & Ryan, 2011; Wang et al., 2013; Qian et al., 2013). For example, McCarthy, Dow & Ryan (2011), investigating the role of Ax21 in *S. maltophilia*, reported that Ax21 secretion depends on RaxB and that Ax21 serves as a cell–cell signaling molecule involved in motility, biofilm formation and virulence. Qian et al. (2013) identified Ax21 in a proteomic study of the *Xoc* RS105 secretome. They showed that deletion of the *ax21* gene resulted in reduced biofilm formation and extracellular-polysaccharide production. They also reported that Ax21 is necessary for full virulence on susceptible hosts. The interaction of this mutant with the XA21 PRR was not tested in this study.

Our interest in the biological function of Ax21, the observation that Ax21 is an *in vitro* substrate for RaxST (Han et al., 2012) (although *in vivo* tyrosine sulfation of Ax21 has not been shown) and the conflicting reports regarding the role of Ax21, motivated us to carry out more detailed studies of the processing and secretion of the Ax21 protein using more sensitive techniques. These recent assays revealed that Ax21 does not require RaxB for processing and is still secreted in *Xoo* strains carrying mutations in *raxA*, *raxB* and *raxC*. We further demonstrate that in *Escherichia coli*, the Ax21 leader sequence is processed by the general secretory pathway and provide evidence that Ax21 is secreted outside the cell as a component of outer membrane vesicles. Finally, we provide genetic data showing that Ax21 is not the activator of XA21-mediated immunity.

## Materials and Methods

### Bacterial strains and growth conditions

*Escherichia coli* strain BL21.DE3 and *Xanthomonas oryzae* pv. *oryzae* (*Xoo*) Philippines race 6 PXO99^A^ (here after called PXO99) were used in this study. Transformed BL21.DE3 cultures were grown in lysogenic broth (LB) with 50 µg/mL kanamycin at 37°C and 230 rpm. PXO99 was cultured on peptone sucrose (PS) agar containing 20 µg/mL cephalexin. The mutant strains PXO99Δ*raxST*, PXO99Δ*raxA*, PXO99Δ*raxB*, PXO99Δ*raxC*, and PXO99Δ*ax21* were described previously (Da Silva et al., 2004; Lee et al., 2009) and cultured in the presence of kanamycin at 50 µg/mL. *Xanthomonas campestris* pv. *campestris* (*Xcc*) strain 33913 and *Xanthomonas euvesicatoria* (*Xe*) strain 85–10 were cultured similarly to PXO99.

For enrichment of Ax21 in the supernatant, cells were grown in 10 mL of yeast extract broth (YEB) media (5 g/L yeast extract, 10 g/L tryptone, 5 g/L NaCl, 5 g/L sucrose, 0.5 g/L MgSO_4_, pH 7.3) to an OD_600_ of ∼1.5, spun down and resuspended in 2 mL of M9 minimal media containing 1.5% glucose and 0.3% casamino acids. Cultures were further incubated at 28°C for 48 h. Then, cells were spun down and the supernatant was passed through a 0.22 µM-filtering unit. The resulting supernatant was enriched for Ax21.

### Rice inoculation, lesion measurements and *in planta* bacterial growth curve analysis

Rice (*Oryza sativa*) TP309 and a transgenic TP309 line carrying the *Xa21* gene driven by its native promoter [TP309-XA21, derivative of line 106-17 (Song et al., 1995)], were germinated in distilled water at 28°C for one week, and then transplanted into soil and grown in 4.5-L pots (4 seedlings/pot) in a greenhouse. Five-to-six-week old plants were transferred to a growth chamber at least two days prior to inoculation. The growth chamber conditions were set to 28°C, 85% humidity and 14/10 day/night cycle. *Xoo* cells were prepared by culturing on PS agar plates with appropriate antibiotics. The cultures were incubated at 28°C for three days. Rice leaves were inoculated with the scissors-clipping method (Kauffman et al., 1973), using cells suspended in distilled water at a density of 10^8^ CFU (colony forming unit)/mL. Lesion lengths were measured 13–14 days after inoculation. The results represent the averages of measurements taken from more than 15 inoculated leaves per strain/plant genotype combination.

For *in planta* bacterial growth analysis, plants were inoculated as described above. At each time point, 6 leaves (from 3 different tillers) were harvested for each bacterial strain/plant genotype combinations. Two leaves from each tiller, representing one biological replicate, were cut to about 2 mm pieces and incubated in 10 mL of water for 2 h at 230 rpm. This suspension was then serially diluted and plated on PSA plates with appropriate antibiotics. Three days later the colonies were counted and the cell number per leaf calculated.

Statistical analysis was carried out using the Tukey-Kramer honestly significant difference test for mean comparison using the JMP software (SAS Institute Inc., Cary, NC, U.S.A.)

### Plasmid construction

The DNA sequence encoding the entire *ax21* gene was amplified from PXO99 genomic DNA and ligated into a modified pET27 vector (Merck KGaA, Darmstadt, Germany) such that the encoded protein has a C-terminal 8x-histidine tag. The sequence cloned included the entire protein with the longer signal peptide MKTSLLALGLLAALPFAASA (see Results and Discussion). Amino acids 18-20, Alanine-Serine-Alanine (the AXA motif), were mutated to Aspartate-Aspartate-Aspartate (DDD) by QuikChange site-directed mutagenesis.

### Western blot

Western blots were carried out following standard procedures (Sambrook Fritsch & Maniatis, 1989). Ax21 protein was detected using an anti-Ax21 antibody. The generation of this antibody was first described in Han et al. (2011), and is described here again. Ax21 monoclonal antibodies were raised in rabbit against the 17 aa Ax21 synthetic peptide (axY ^S^22). Peptides and antibodies were generated by Pacific Immunology (http://www.pacificimmunology.com/). For Western blots, the Ax21 antibody and a secondary anti-rabbit IgG antibody were used at dilutions of 1:3,000 and 1:5,000, respectively. Western blots detecting the His tag were performed using anti-His (Qiagen 34660) and anti-mouse IgG antibodies at dilutions of 1:2,000.

### PXO99 supernatant fractionation and proteinase K treatment

To separate the outer membrane vesicle (OMVs) fraction from cell-free supernatants ultracentrifugation was applied at ∼180,000 g for 2 h. The resulting membrane pellet was resuspended in 200 µL of water. Proteinase K treatment was carried out by incubating 40 µL of the OMVs pellet with 2 µg of proteinase K at 37°C for 30 min.

## Results and Discussion

### Ax21 secretion does not require *raxA*, *raxB*, or *raxC*

**Figure 1:**
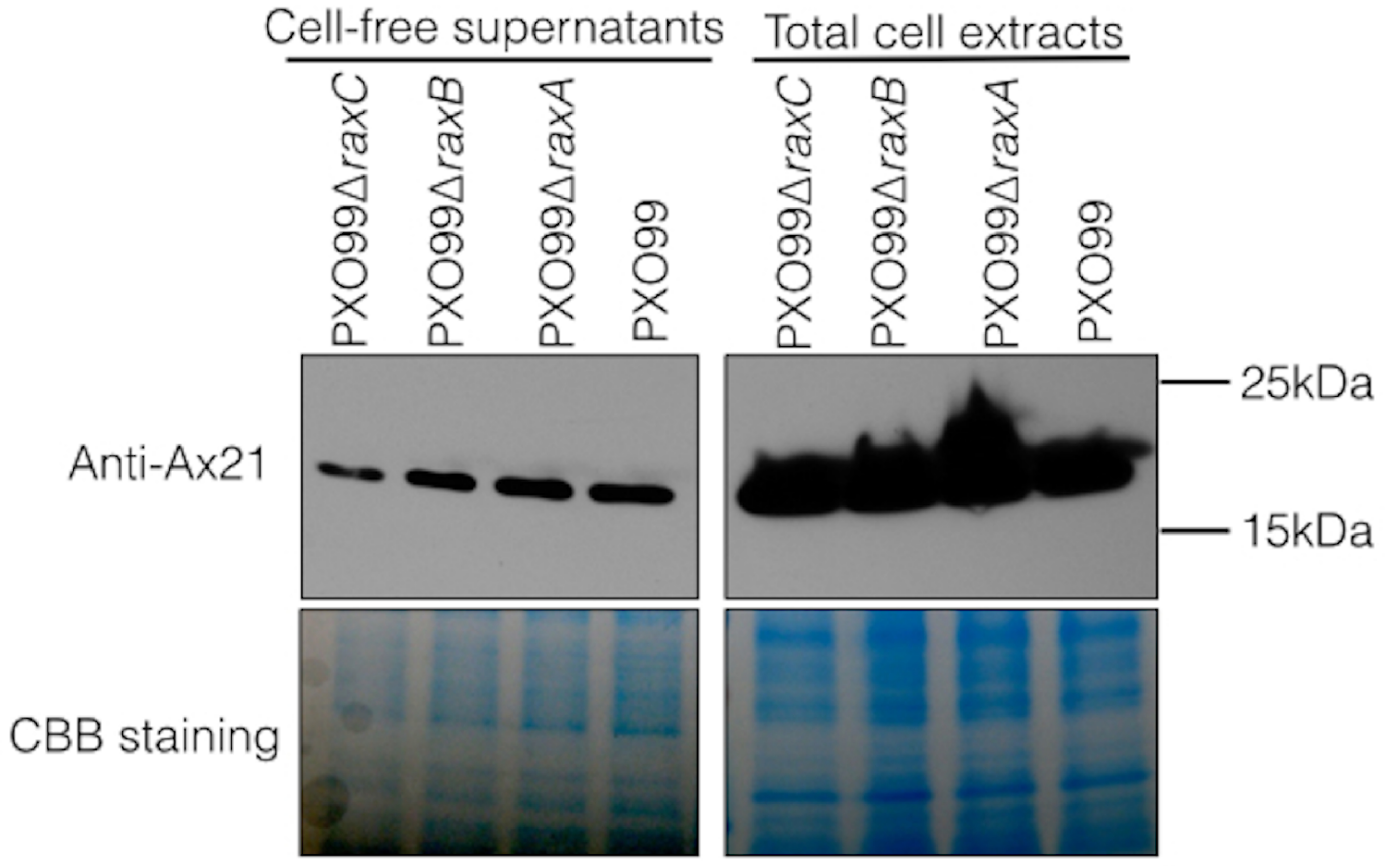
Ax21 secretion does not depend on RaxABC. Western blot analysis, using an anti-Ax21 antibody (upper panel), was carried out on total cells and cell-free supernatants of wild type strain PXO99 and the PXO99Δ*raxA*, PXO99Δ*raxB* and PXO99Δ*raxC* knockout strains grown under Ax21-enriching conditions (see material and methods). Similar levels of Ax21 are detected in supernatants of all tested strains. In all strains Ax21 is of similar size (predicted size of a mature, processed Ax21 protein is ∼19 kDa). Loading control with Coomassie brilliant blue staining (CBB) is presented in the lower panel.

In earlier experiments, we assayed for the presence of Ax21 in supernatants of wild type and the *raxA* and *raxC* mutant strains using silver staining of PAGE gels (Lee et al., 2009). To more carefully assess Ax21 secretion in these mutant strains, we used antibodies generated against Ax21. We carried out Western blot analysis to validate the presence of secreted Ax21 in the extracellular milieu. PXO99 cultures were grown similarly to the previously described methods for enriching for Ax21 in the supernatant (Han et al., 2011), and probed with an anti-Ax21 antibody (Fig. 1). As reported previously, Ax21 is detected in the supernatants of PXO99 (Lee et al., 2009). However, using this more sensitive and specific detection method, we found that Ax21 is also present in the supernatants of PXO99Δ*raxA*, PXO99Δ*raxB* and PXO99Δ*raxC* strains (Fig. 1). These results indicate that RaxA, RaxB and RaxC are not required for Ax21 secretion. These experiments also show that the Ax21 protein is of the mature size in all the samples, including PXO99Δ*raxB*, indicating that Ax21 does not require the presence of RaxB for processing, as was previously hypothesized (Lee et al., 2009; Lee et al., 2013).

We next tested if Ax21 is present in supernatants of *Xcc*. The rationale for this experiment is that *Xcc* lacks predicted orthologs of RaxA and RaxB. Thus, if Ax21 is secreted in *Xcc*, it would indicate that these genes are not required for its secretion. We found that Ax21 is present in *Xcc* supernatants grown under enriching conditions (Fig. S1). Together, these experiments indicate that Ax21 is not processed by RaxB and does not require the predicted type I secretion components (RaxA, RaxB and RaxC) for secretion as previously suggested (Lee et al., 2009; Lee et al., 2013; Han et al., 2011; Han et al., 2013).

These results conflict with publications by two independent research groups. The first, by McCarthy, Dow & Ryan (2011), showed that Ax21 activity from supernatants of *S. maltophilia* strain K279a carrying a mutation in a presumed raxB ortholog (Smlt2001) is reduced compared with that of supernatants from the wild type strain. From this experiment the authors concluded that RaxB is partially required for Ax21 secretion. However, our analysis of Smlt2001 reveals that it lacks the unique N-terminal peptidase domain (C39, residues 17-143 in the PXO99A genome annotation), characteristic of the RaxB ABC-transporter. Instead, Smlt2001 shows a much higher similarity to PXO_01193, a PXO99 ABC-transporter that lacks the N-terminal C39 peptidase domain. This analysis suggests that Smlt2001 is not an ortholog of RaxB. We were not able to identify a predicted RaxB ortholog in the *S. maltophilia* K279a genome. The second publication, by Wang et al. (2013), reported that Ax21 is not secreted in the absence of RaxA in Xoc. We have not tested the role of RaxA in Xoc.

### Ax21 is processed by the general secretory (Sec) system

Many secreted proteins have N-terminal leader sequences, which are cleaved during export. We have shown through silver staining and Western blot analyses of *Xoo* supernatants that the size of Ax21 secreted from PX099 corresponds to the size of the mature, processed protein. MS/MS analysis of the excised band reveals that peptides corresponding to Ax21 lack the N-terminal region. These results indicate that the N-terminal region of Ax21 is cleaved before or during secretion (Lee et al., 2009; Lee et al., 2013). The involvement of RaxB, an ABC-transporter containing a peptidase domain, in triggering XA21-mediated immunity, led us to the hypothesis that RaxB processes Ax21 before or during secretion. RaxB carries an N-terminal C39 peptidase domain, which is predicted to recognize and cleave GG-leader peptides (Da Silva et al., 2004). Such leader sequences typically have the motif LSX_2_ELX_2_IXGG (where X can be any amino acid) with cleavage occurring immediately after the double glycine (Dirix et al., 2004). These are called GG-type or double glycine leader peptides. Ax21 lacks this motif.

Because RaxB does not process Ax21 (Fig. 1), and Ax21 lacks the GG-leader motif, we investigated other mechanisms of processing. Reanalysis of the Ax21 leader sequence revealed a signal peptide that typically targets proteins to the general secretory (Sec) pathway. The Sec system is the primary mechanism by which proteins are delivered across the microbial cytoplasmic membrane (Natale, Brüser & Driessen, 2008). Sec-targeted proteins are delivered to the primary Sec machinery by the secretion specific chaperone SecB (Natale, Brüser & Driessen, 2008). The protein is translocated across the membrane through the heterotrimeric integral membrane complex SecYEG driven by the associated molecular motor SecA. The signal peptide is cleaved from the protein during or shortly after translocation by a membrane bound signal peptidase.

We previously reported that secreted Ax21 is missing the first 15 amino acids MLALGLLAALPFAASA (Lee et al., 2009). This sequence is based on the annotation of the PXO99 genome (Salzberg et al., 2008). This annotation of the *ax21* gene begins translation with the alternate start codon TTG. However, examination of the genomic DNA sequence suggests that translation likely begins at a typical ATG start codon 12 base pairs upstream. Moreover, a putative Shine-Dalgarno Sequence (AGG) is positioned 10 bp upstream of the ATG codon. Based on this analysis, we predict that the correct leader sequence is **MKTS**LLALGLLAALPFAASA. Most of the Ax21 homologs from other strains and species are annotated with similar longer leader sequences.

**Figure 2:**
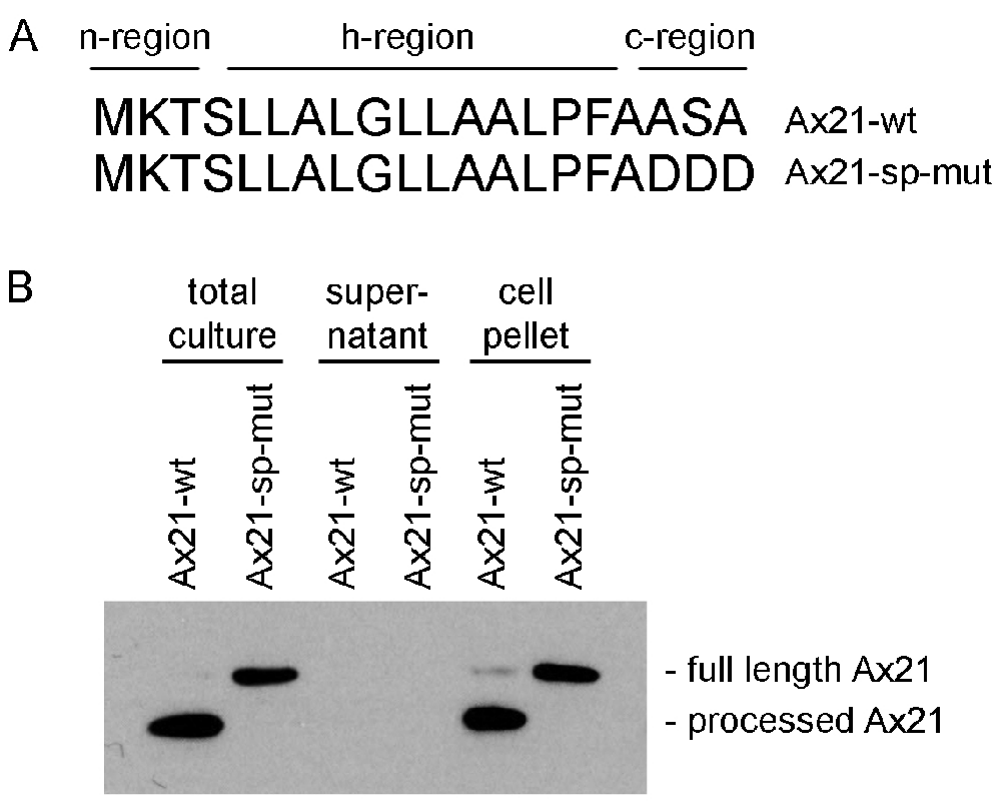
Ax21 is targeted to the Sec pathway. (A) The first 20 amino acids of Ax21 are predicted to constitute a Sec signal peptide containing a positively charged polar n-region, a hydrophobic central region, and a c-region containing the leader peptidase motif AXA. (B) Recombinant Ax21 was expressed in BL21.DE3 *E. coli* with a C-terminal His tag. Samples from total cultures, cell-free supernatants, and resuspended cell pellets were analyzed by SDS-PAGE followed by Western blot analysis. An anti-His antibody was used to detect the C-terminal His tag. Processed Ax21-His (21 kDa) is present in *E. coli* cells, but is not detected in the supernatant. When the leader peptidase motif AXA is mutated to DDD (Ax21-sp-mut), the protein is not processed (23 kDa).

The program SignalP 4.1, which predicts the presence of classical, but not GG-type signal peptides, indicates that both the annotated shorter sequence (16 amino acids) and the longer sequence (20 amino acids) are predicted signal peptides (Fig. 2A) (Petersen et al., 2011). The Ax21 leader sequence has the hallmarks of a typical Sec signal peptide as shown in Fig. 2A. These include a positively charged N-terminus (n-region), a hydrophobic core (h-region), and a short C-terminal region containing the motif AXA (Natale, Brüser & Driessen, 2008). The leader peptidase cleaves the protein immediately after the AXA motif. This corresponds with the region that was found to be absent from extracellular Ax21 (Lee et al., 2009).

To test if Ax21 is processed by the Sec system and not by RaxB, we expressed recombinant C-terminal His-tagged Ax21 in *E. coli* (BL21.DE3), a strain that has no homologs of RaxB. We analyzed Ax21 processing by Western blot analysis, probing for the C-terminal His-tag. We observed that in total cell cultures and in cell pellets the N-terminus of Ax21 was processed (Fig. 2B). When the predicted ASA cleavage motif was mutated, Ax21 was no longer processed (Fig. 2B). Ax21 was not detected in *E. coli* supernatants probably since the conditions used for expression were not set for enriching Ax21 in the supernatant (see below and Fig. S4). Altogether, these results indicate that Ax21 possesses a typical Sec signal peptide, which is processed by the Sec machinery.

### Ax21 secretion is associated with outer membrane vesicles

Not all proteins targeted to the Sec pathway are secreted. In Gram-negative bacteria, proteins processed by the Sec system can be destined to the inner membrane, periplasmic space, outer membrane, or the extracellular space. We previously showed that Ax21 was found in the supernatant of *Xoo* cultures, and culturing methods were developed for Ax21 enrichment in the supernatant (Lee et al., 2009; Han et al., 2011).

PFAM searches of the Ax21 sequence suggest that it falls into the family of β-barrel outer membrane proteins (OMP) (e-value = 9.1e−08) (Altschul et al., 1990; Sonnhammer, Eddy & Durbin, 1997). The β-barrel topology prediction servers PRED-TMBB (Bagos et al., 2004) and BOCTOPUS (Hayat & Elofsson, 2012) predict that Ax21 forms a 10-stranded β-barrel (Fig. S2). There are several known 10-stranded beta barrel proteins with solved three-dimensional structure (Vandeputte-Rutten et al., 2001; Prince, Achtman & Derrick, 2002; Eren et al., 2010). However, these have very low amino acid sequence similarity to Ax21. Using the automated homology-modeling program I-TASSER, Ax21 is predicted to form an 8-stranded β-barrel structure. Despite the variation between these prediction tools, all three show that Ax21 has clear features of an outer-membrane protein with a β-barrel structure (Koebnik, Locher & Van Gelder, 2000).

**Figure 3:**
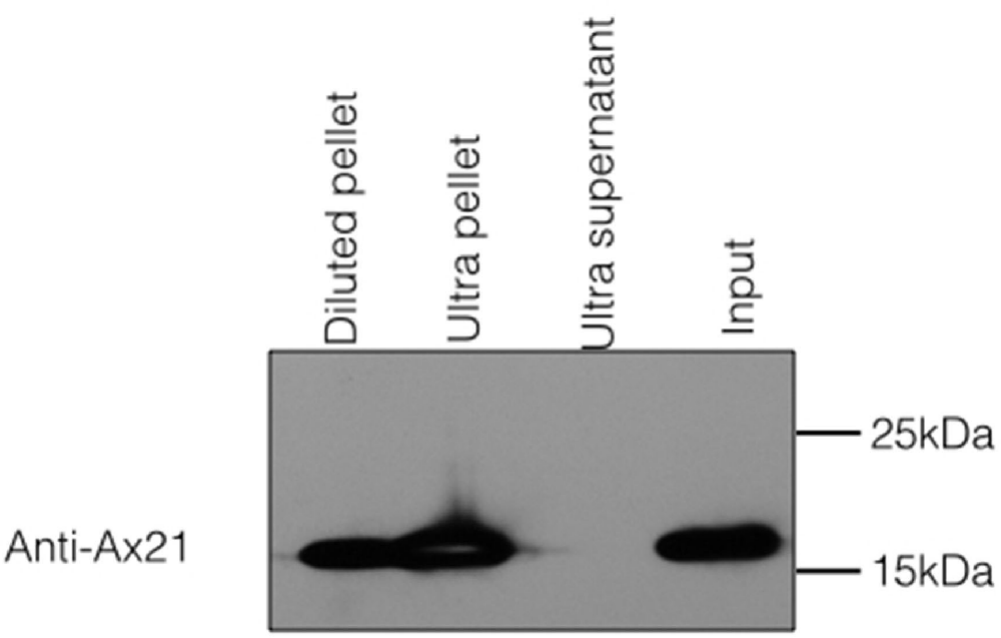
Ax21 is an outer membrane protein secreted in association with outer membrane vesicles (OMVs). *Xoo* cell-free supernatants were centrifugation at 180,000 g for 2 h to separate OMVs from soluble proteins. Fractions were then subjected to Western blot analysis with an anti-Ax21 antibody. Input: cell-free supernatant before centrifugation, ultra supernatant: supernatant after ultracentrifugation, ultra pellet: OMVs pellet after ultracentrifugation that was resuspended in 200 µL of water, diluted pellet: pellet sample diluted back to the original volume of the input sample. This figure shows that Ax21 is present in cell-free supernatants exclusively in an insoluble form in membrane vesicles. This experiment was repeated twice with similar results.

Because Ax21 is found in supernatants and due to its predicted β-barrel structure, we hypothesized that secreted Ax21 is embedded in outer membrane vesicles (OMVs). OMVs are spherical structures derived from the budding of sections of the outer membrane (McBroom & Kuehn, 2007). To test this hypothesis, we generated *Xoo* supernatants using the previously described method for enrichment of Ax21. The enrichment method used in our previous studies to purify Ax21 required growing *Xoo* to a high concentration, spinning cells down, removing the supernatant and resuspending the cell pellet in a small volume of minimal media. The concentrated culture was further incubated for 24–48 h. The cells were pelleted by centrifugation, and the supernatant was filtered through 0.22 µM filter. To separate the OMVs from soluble, secreted proteins, the cell-free supernatant was ultracentrifuged at ∼180,000 g for 2 h. The resulting supernatant and OMV fractions were probed for the presence of Ax21 by Western blot. As shown in Fig. 3, Ax21 can be detected in the enriched supernatant; however, it was absent from the supernatant following ultracentrifugation. In contrast, the OMVs pellet had a strong band representing Ax21, indicating that Ax21 in the cell-free supernatant was associated with OMVs. These results further suggest that Ax21 is not a soluble secreted protein, but rather an outer membrane protein secreted via the OMVs secretory pathway.

To test whether the secretion of Ax21 via the OMVs pathway is conserved in different *Xanthomonas* stains, we examined OMVs of *Xcc* and *Xanthomonas euvesicatoria* (*Xe*). These strains were chosen as representatives of strains lacking or containing RaxA/B orthologs, respectively. Similar to *Xoo*, Ax21 was associated exclusively with the OMV pellets of both strains (Fig. S1), suggesting that the secretion of Ax21 via the OMV secretory pathway is conserved among at least 3 different *Xanthomonas* species and is independent of the RaxA/B components.

To test whether Ax21 is embedded in membrane vesicles, or merely associated with them, isolated OMVs were treated with proteinase K and then analyzed by Western blot. While the majority of the OMV proteins appeared to be degraded, Ax21 was not (Fig. S3). Adding the Triton X-100 detergent caused a slight decrease in the intensity of the Ax21 band. These results indicate that Ax21 is embedded in the outer membrane and are consistent with the homology and structural prediction, which suggest that Ax21 is an OMP.

OMVs production is increased as a response to cellular stress (McBroom & Kuehn, 2007). This may explain why more Ax21 is found in the supernatant when *Xoo* is grown using the enrichment method as described above (Fig. S4). The high cell concentration and lack of nutrients may induce stress responses in the cells leading to elevated levels of OMVs production. Ax21 is not detected in supernatants from *Xoo* grown normally in rich media unless the supernatants are highly concentrated (Fig. S4).

### Ax21 is not required for activation of XA21-mediated immunity

The findings that Ax21 is an OMP, processed by the Sec system and released in OMVs, does not fall in line with our previous findings and proposed model that Ax21 is a RaxA/B secreted activator of XA21 mediated immunity. We had previously reported that a PXO99Δ*ax21* insertion mutant strain could overcome XA21-mediated immunity (Lee et al., 2009; Lee et al., 2013). However, a careful examination of our collection of bacterial mutant strains revealed that several strains were mixed up or had the incorrect genetic profile (Fig. S5) (Ronald, 2013).

**Figure 4:**
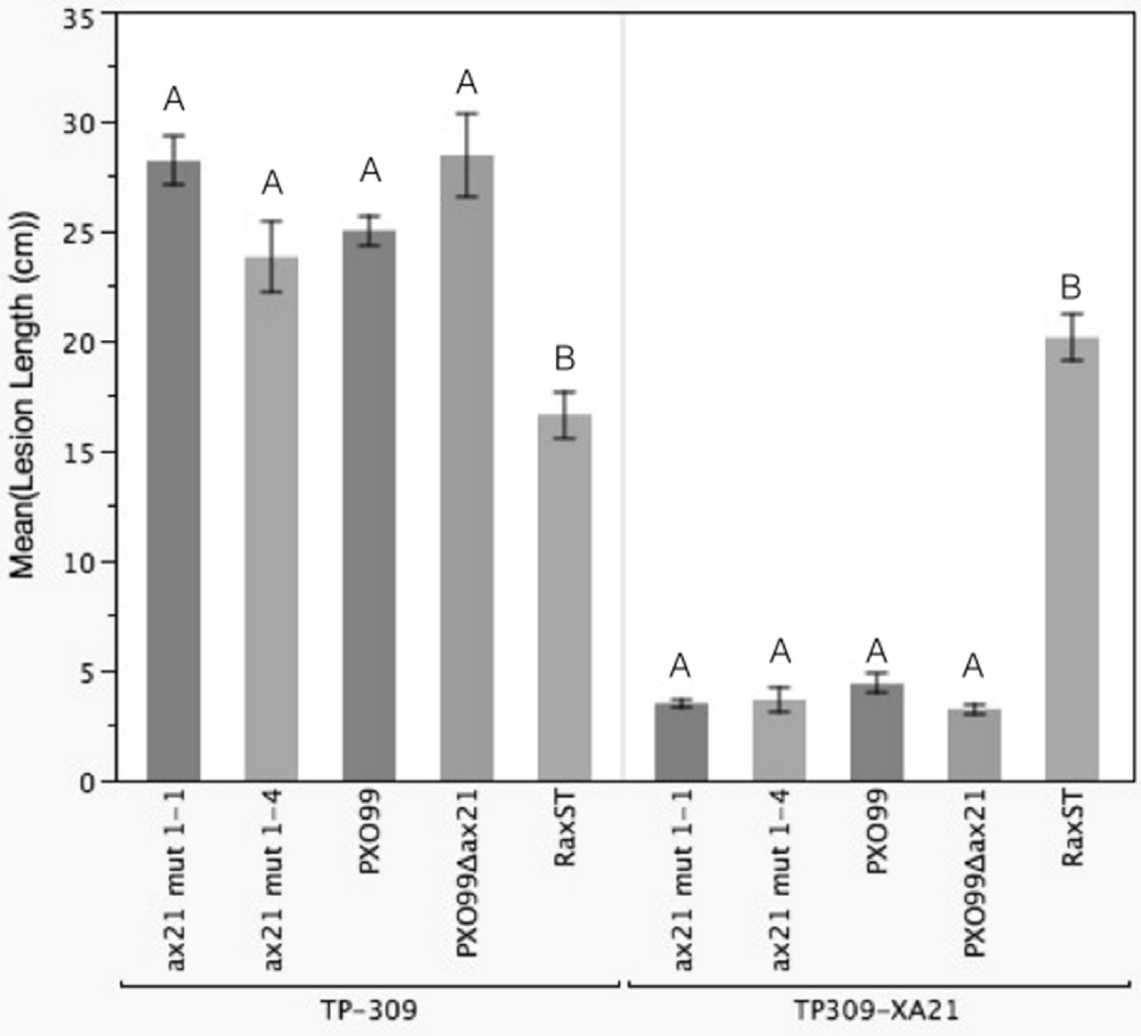
*Ax21* insertion mutant strains retain the ability to activate XA21-mediated immunity. To re-examine whether the *ax21* mutant strain can evade XA21-mediated immunity, we used a validated PXO99Δ*ax21* (see Fig. S5) to inoculate rice using a standard leaf-clipping assay. We have also added two newly generated *ax21* insertion mutant strains labeled ax21 mut1-1 and ax21 mut1-4. PXO99 and PXO99Δ*raxST* were used as control strains on XA21-expressing plants. Disease lesions were scored 13–14 days post inoculation. Bars represent averages of at least 15 leaves ± SE. Statistical analysis was done using the Tukey-Kramer honestly significant difference test for mean comparison using the JMP software. Different letters within plant genotype, represent significant differences (*P* < 0.01) within each plant genotype.

We identified a correct PXO99Δ*ax21* mutant strain in the collection, which we validated by PCR, Southern blot and Western blot (Fig. S5). The correct mutant had a kanamycin resistance cassette inserted near the C-terminus of the gene by double homologous recombination. We inoculated TP309 and TP309-XA21 with the validated mutant using the scissor clipping method. As shown in Fig. 4, PXO99Δ*ax21* forms short lesions on TP309-XA21 rice, similarly to the wild type strain, while PXO99Δ*raxST* forms long water-soaked lesions as previously reported (Da Silva et al., 2004). These results were further supported by an *in planta* growth curve analysis, where no statistical differences in bacterial burden could be observed between the wild type strain PXO99 and the PXO99Δ*ax21* throughout the time course (Fig. S6). To confirm this finding, we generated two additional *ax21* mutant strains, using two different knockout plasmids. These mutants had identical *in planta* phenotype to the PXO99Δ*ax21* strain (Fig. 4). These results indicate that the PXO99Δ*ax21* mutant does not evade XA21-mediated immunity as previously reported (Lee et al., 2009; Lee et al., 2013).

## Conclusions

We report here that Ax21 is an outer membrane protein processed by the Sec system and secreted via the outer membrane vesicle secretory pathway. Additionally, we show that strains carrying mutations in the *ax21* gene (PXO_03968) still activate XA21-mediated immunity (Lee et al., 2013). These results indicate that Ax21 is not processed and secreted via the RaxABC secretion system and is likely not the activator of XA21-mediated immunity. Additional research is needed to determine the biological function of this protein.

## Supplemental Information

10.7717/peerj.242/supp-1 Figure S1 Ax21 secretion via OMVs is conserved in *X. euvesicatoria* and *Xcc*. Both strains were grown in Ax21-enriching conditions as described for *Xoo*, and cell-free supernatants were centrifuged at 180,000 g for 2 h to pellet OMVs. Samples were then subjected to Western blot analysis with an anti-Ax21 antibody. Input: cell-free supernatant before centrifugation, ultra supernatant: supernatant after ultracentrifugation, ultra pellet: OMVs pellet after ultracentrifugation that was resuspended in 200 mL of water. Ax21 is absent from supernatants after ultracentrifugation indicating that it is in the insoluble fraction of OMVs.

10.7717/peerj.242/supp-2 Figure S2 Topology prediction of Ax21. (A) Predicted membrane topology from BOCTOPUS (Hayat & Elofsson, 2012). Predicted transmembrane β-strands are shown in grey; inner membrane loops are in red; outer membrane loops are in blue. (B) Two-dimensional rendering of the predicted Ax21 topology from PRED-TMBB. For both (A) and (B), the numbering begins from the first amino acid of the processed Ax21.

10.7717/peerj.242/supp-3 Figure S3 Ax21 is embedded in the outer membrane. OMVs were purified as described in Materials and Methods and were then treated with Proteinase K and/or 0.1% Triton X-100 for 30 min. While most proteins in the OMVs preparation were degraded by proteinase K, as can be seen in the “Pro K”- treated lanes (CBB straining, left panel), Ax21 remained at the same level as in non-treated samples (Western blot, right panel), indicating that it is embedded in the outer membrane. Some degradation of Ax21 can be observed only when proteinase K treatment was combined with the Triton X-100 detergent.

10.7717/peerj.242/supp-4 Figure S4 Ax21 secretion is enhanced under enrichment conditions. PXO99 was grown under regular, or enrichment conditions and tested for Ax21 presence in the cell-free supernatants. Under regular conditions, PXO99 was grown until an OD_600_ of ∼2.0 in YEB, then the cells were pelleted by centrifugation. The supernatant was filtered using 0.22 mM filter (supernatant). This sample was further concentrated x10 using a 3kDa Centricon. The enrichment sample was prepared as described before and supernatant was concentrated in the same manner as for YEB. Western blot analysis was done using the anti-Ax21 antibody.

10.7717/peerj.242/supp-5 Figure S5 Validation of the PXO99Δ*ax21* insertion mutant. The *ax21* insertion mutant described in this paper (named here PXO99Δ*ax21*-2) and a mislabeled *ax21* mutant strain in our collection (named here PXO99Δ*ax21*-1) were tested by (A) PCR, (B) Southern blot and (C) Western blot analyses. (A) PCR primers for the full-length *ax21* ORF were used (forward primer- CGC**CATATG**AAGACTTCTTTGCTGGCCCT; reverse primer- CGC**GGATCC**TTACCAGCTGAAGCGCGG; (in bold are sequences for restriction enzymes used for cloning). PCR of the *ax21* gene is expected to yield a ∼0.6 kb product from PXO99, and a ∼1.8 kb from the ax21 insertion mutant (includes ∼1.2 kb insertion of the kanamycin resistance gene). (B) Southern blot analysis was carried on out on *MscI*-digest genomic DNA probed with a labeled 1.2 kb kanamycin resistance gene. The predicted size for a correct *ax21* insertion mutant is 4227 bp. (C) Western blot analysis on total cells using the anti-Ax21 antibody as described in Material and Methods section. For each assay the wild type PXO99 strain, the PXO99Δ*ax21*-2 insertion mutant strain showing the correct *DNA* profile and the mislabeled PXO99Δ*ax21*-1 strain showing an incorrect DNA profile are shown.

10.7717/peerj.242/supp-6 Figure S6 *In planta* bacterial growth curve analysis shows that the PXO99Δ*ax21* validated insertion mutant is not virulent on TP309-XA21. Bacterial growth *in planta* was assessed as described in the Materials and Methods section. When inoculated on the TP309-XA21 rice line (right panel), PXO99Δ*ax21* does not grow to higher levels than PXO99, while the control, XA21-virulent strain PXO99Δ*raxST*, grows to significantly higher numbers than both PXO99 and PXO99Δ*ax21* at 8 and 12 days after inoculation (dai). On TP309 (right panel) the PXO99Δ*raxST* shows lower cell titer only at the 12 dai point. Each time point represents an average of 6 leaves ± SE. Statistical analysis was done for each time point using the Tukey-Kramer HSD test. Asterisk represents significant difference at *p* < 0.05.
