# Supplementary figures and images for: The Xanthomonas Ax21 protein is processed by the general secretory system and is secreted in association with outer membrane vesicles
Source: PeerJ. 2014 Jan 7;2:e242. doi: 10.7717/peerj.242 (PMC3897388; doi:10.7717/peerj.242)

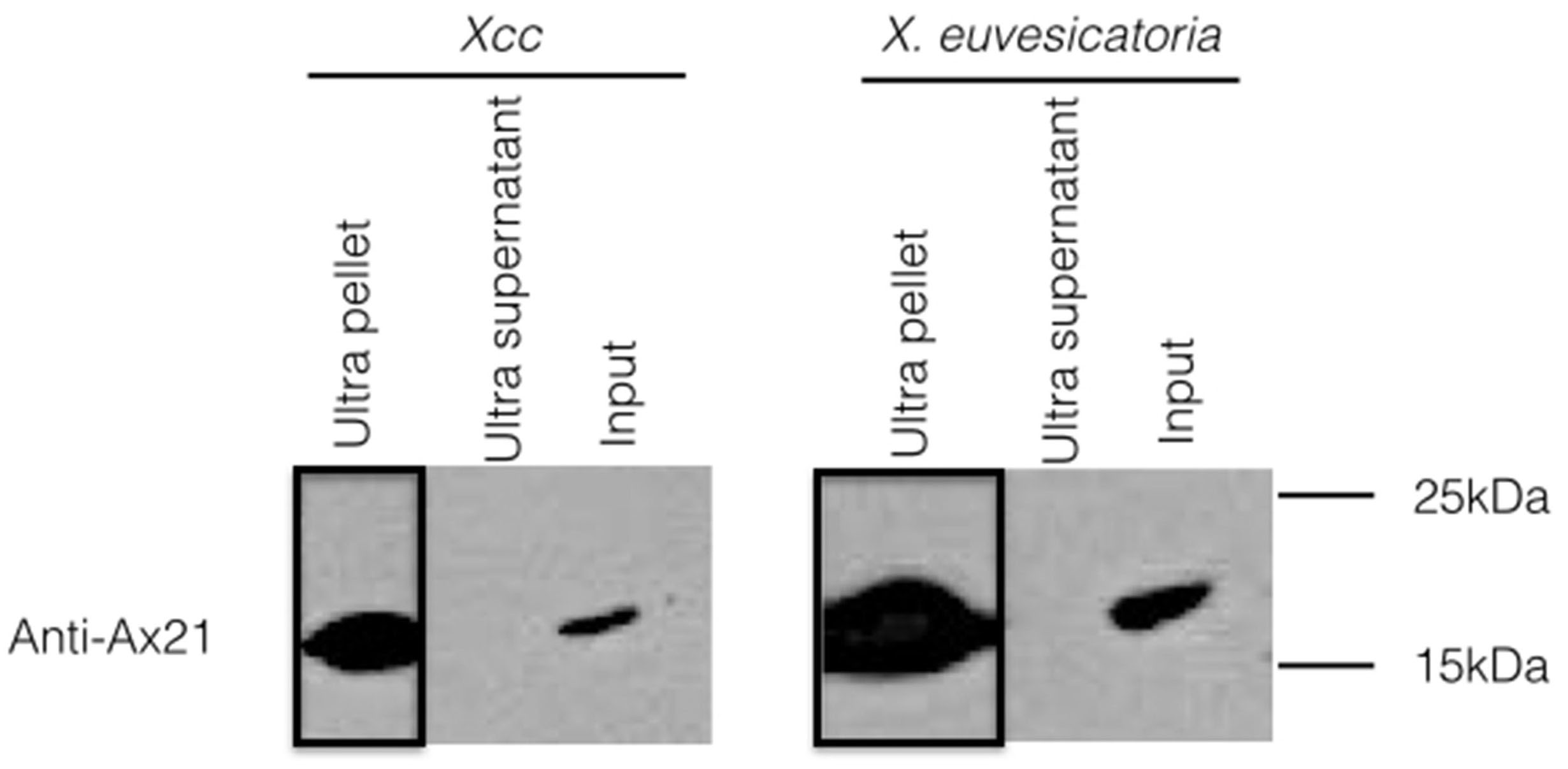

Supplement: Figure S1 — Both strains were grown in Ax21-enriching conditions as described for Xoo, and cell-free supernatants were centrifuged at 180,000 g for 2 h to pellet OMVs. Samples were then subjected to Western blot analysis with an anti-Ax21 antibody. Input: cell-free supernatant before centrifugation, ultra supernatant: supernatant after ultracentrifugation, ultra pellet: OMVs pellet after ultracentrifugation that was resuspended in 200 mL of water. Ax21 is absent from supernatants after ultracentrifugation indicating that it is in the insoluble fraction of OMVs. [file peerj-02-242-s001.png]

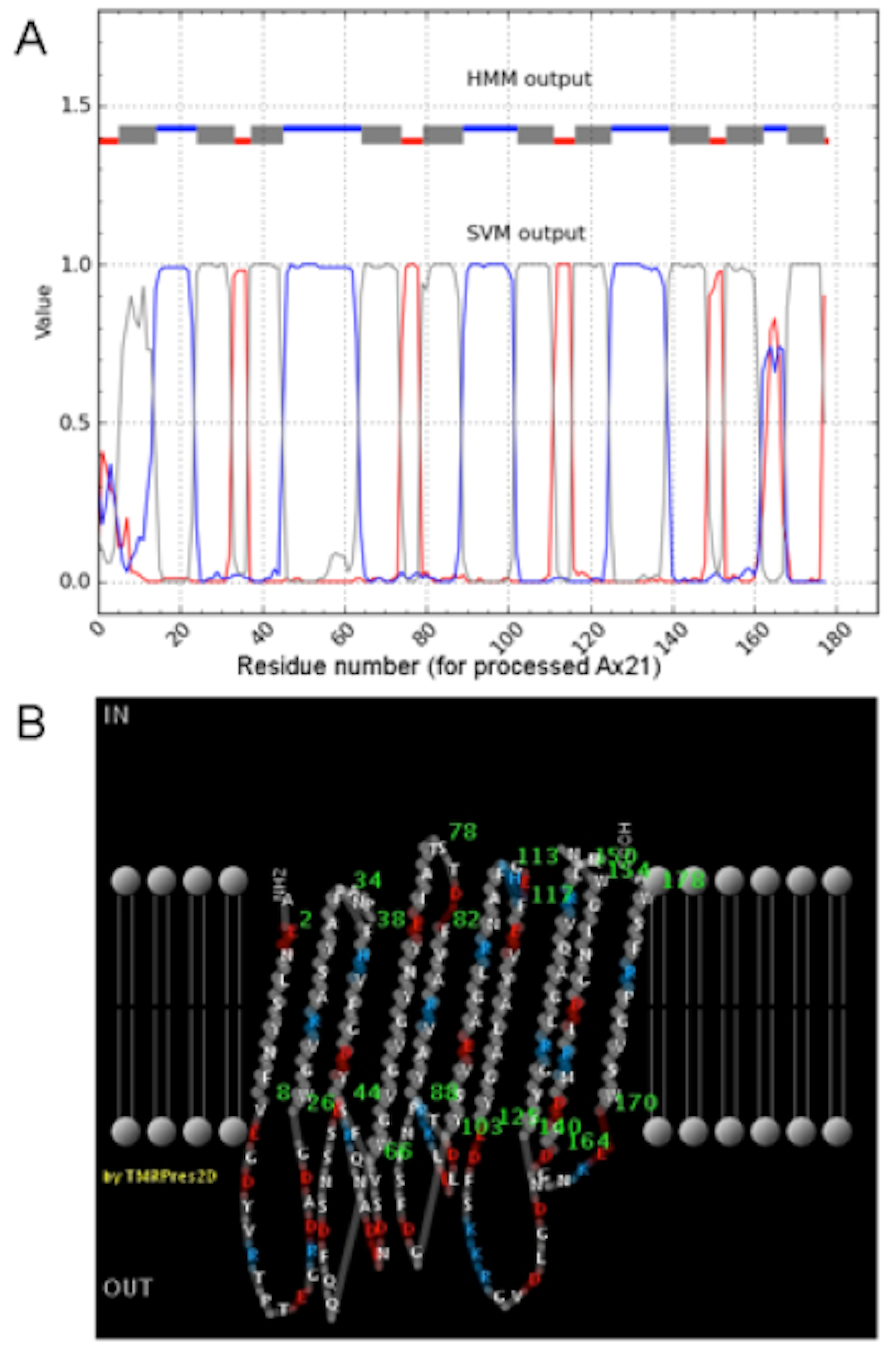

Supplement: Figure S2 — (A) Predicted membrane topology from BOCTOPUS (Hayat & Elofsson, 2012). Predicted transmembrane β-strands are shown in grey; inner membrane loops are in red; outer membrane loops are in blue. (B) Two-dimensional rendering of the predicted Ax21 topology from PRED-TMBB. For both (A) and (B), the numbering begins from the first amino acid of the processed Ax21. [file peerj-02-242-s002.png]

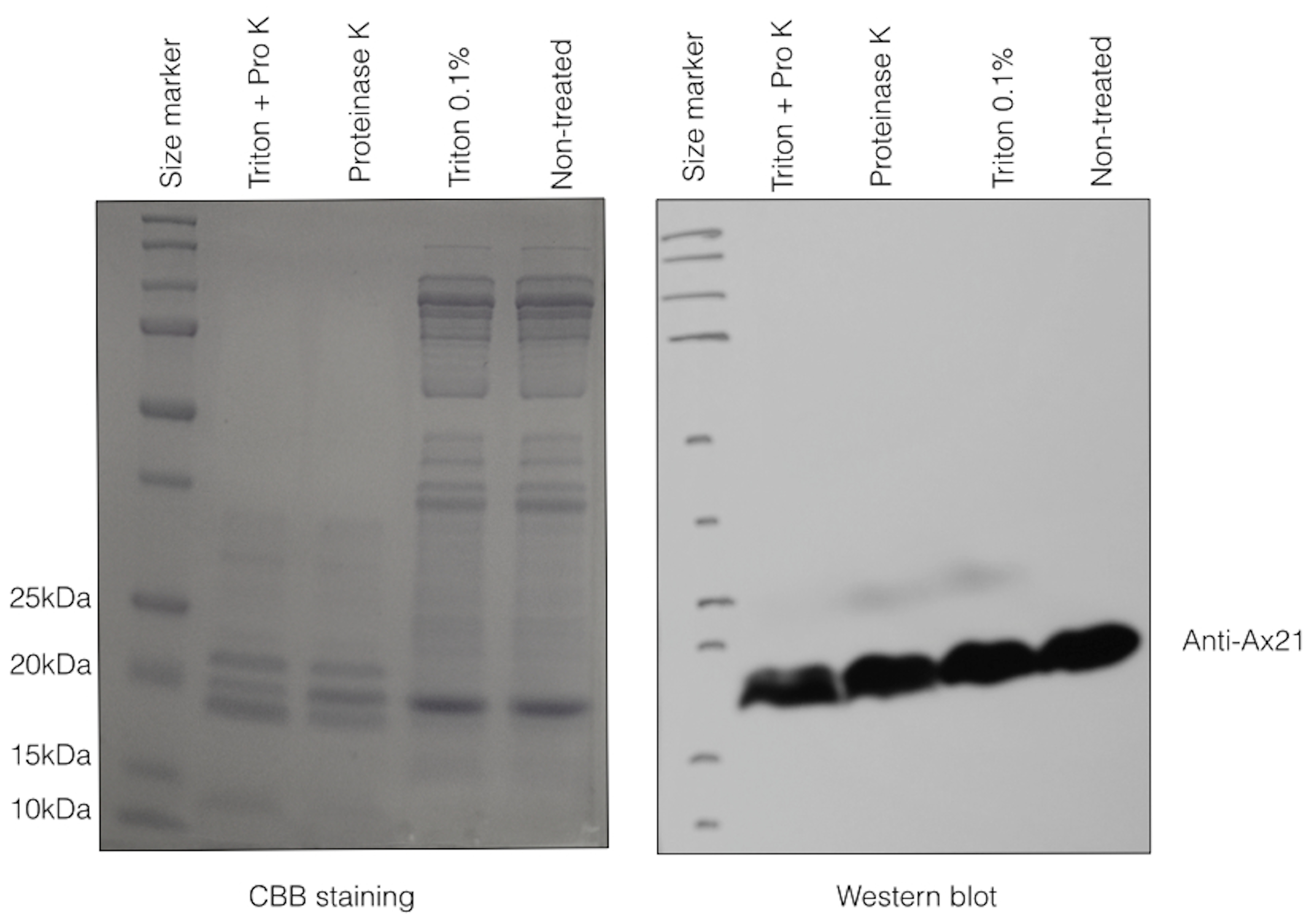

Supplement: Figure S3 — OMVs were purified as described in Materials and Methods and were then treated with Proteinase K and/or 0.1% Triton X-100 for 30 min. While most proteins in the OMVs preparation were degraded by proteinase K, as can be seen in the “Pro K”- treated lanes (CBB straining, left panel), Ax21 remained at the same level as in non-treated samples (Western blot, right panel), indicating that it is embedded in the outer membrane. Some degradation of Ax21 can be observed only when proteinase K treatment was combined with the Triton X-100 detergent. [file peerj-02-242-s003.png]

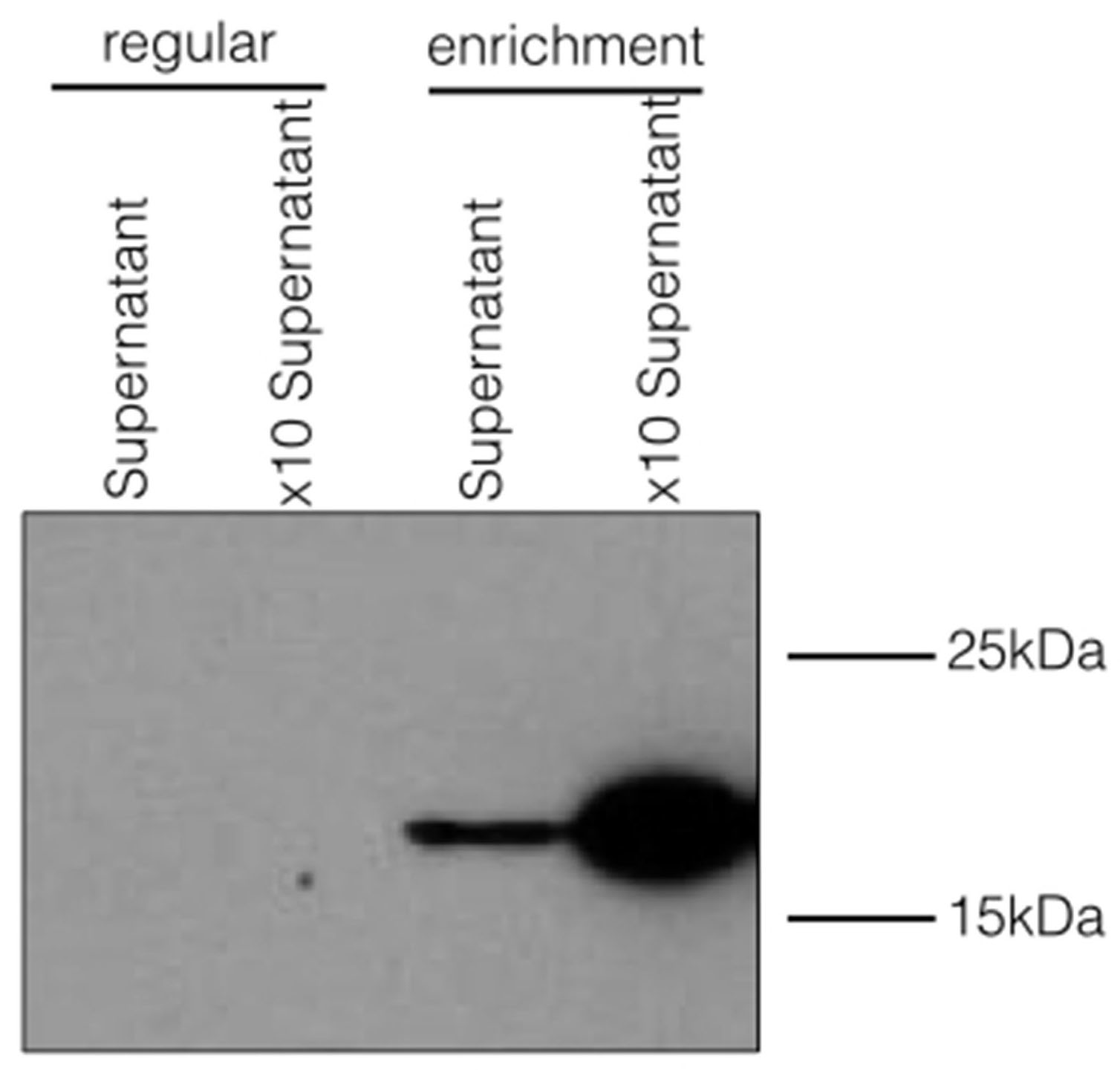

Supplement: Figure S4 — PXO99 was grown under regular, or enrichment conditions and tested for Ax21 presence in the cell-free supernatants. Under regular conditions, PXO99 was grown until an OD600 of ∼2.0 in YEB, then the cells were pelleted by centrifugation. The supernatant was filtered using 0.22 mM filter (supernatant). This sample was further concentrated x10 using a 3kDa Centricon. The enrichment sample was prepared as described before and supernatant was concentrated in the same manner as for YEB. Western blot analysis was done using the anti-Ax21 antibody. [file peerj-02-242-s004.png]

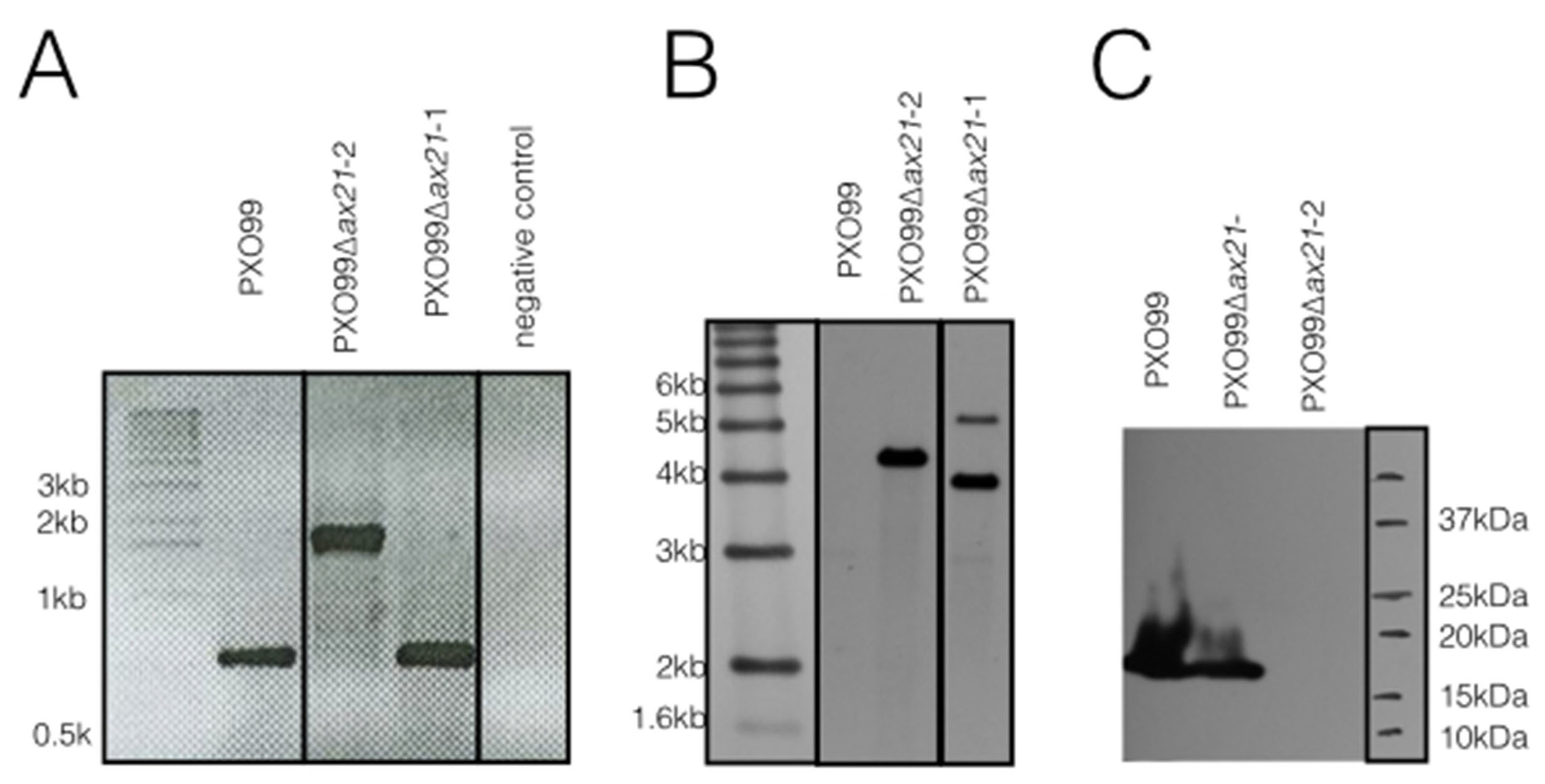

Supplement: Figure S5 — The ax21 insertion mutant described in this paper (named here PXO99Δax21-2) and a mislabeled ax21 mutant strain in our collection (named here PXO99Δax21-1) were tested by (A) PCR, (B) Southern blot and (C) Western blot analyses. (A) PCR primers for the full-length ax21 ORF were used (forward primer- CGCCATATGAAGACTTCTTTGCTGGCCCT; reverse primer- CGCGGATCCTTACCAGCTGAAGCGCGG; (in bold are sequences for restriction enzymes used for cloning). PCR of the ax21 gene is expected to yield a ∼0.6 kb product from PXO99, and a ∼1.8 kb from the ax21 insertion mutant (includes ∼1.2 kb insertion of the kanamycin resistance gene). (B) Southern blot analysis was carried on out on MscI-digest genomic DNA probed with a labeled 1.2 kb kanamycin resistance gene. The predicted size for a correct ax21 insertion mutant is 4227 bp. (C) Western blot analysis on total cells using the anti-Ax21 antibody as described in Material and Methods section. For each assay the wild type PXO99 strain, the PXO99Δax21-2 insertion mutant strain showing the correct DNA profile and the mislabeled PXO99Δax21-1 strain showing an incorrect DNA profile are shown. [file peerj-02-242-s005.png]

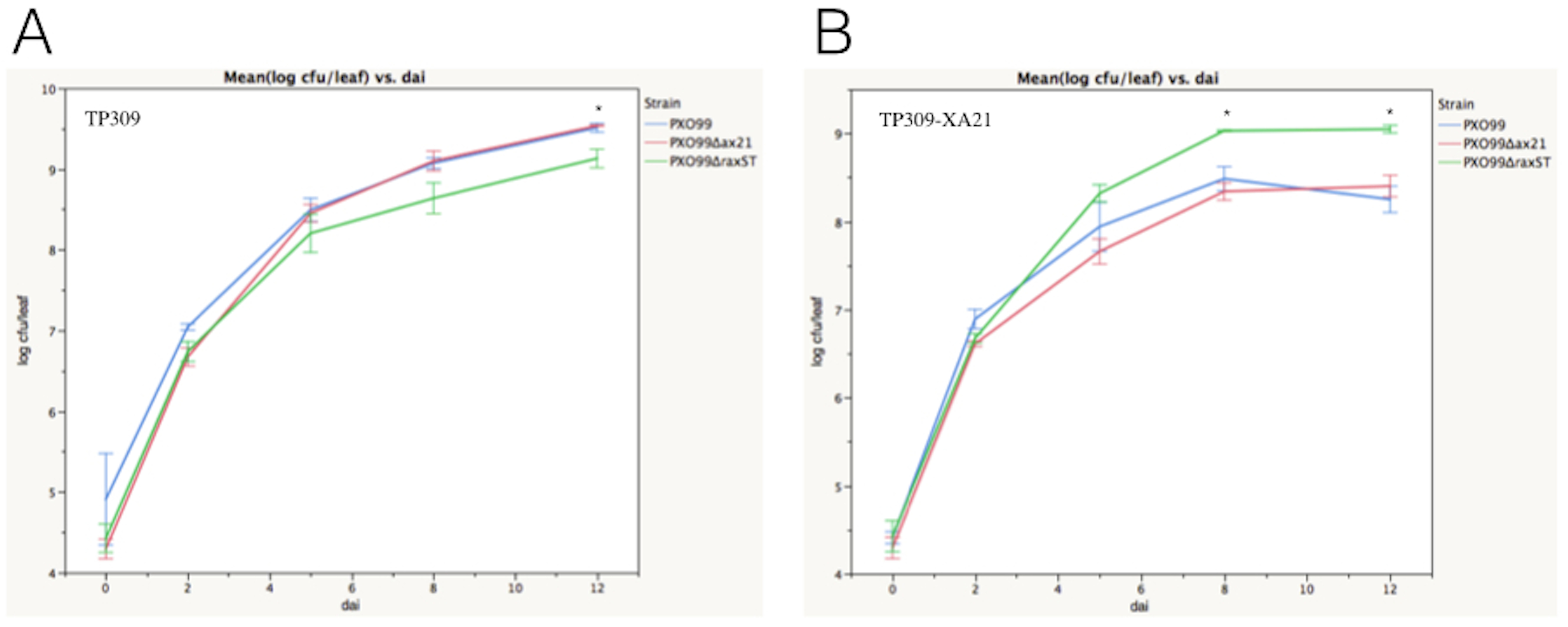

Supplement: Figure S6 — Bacterial growth in planta was assessed as described in the Materials and Methods section. When inoculated on the TP309-XA21 rice line (right panel), PXO99Δax21 does not grow to higher levels than PXO99, while the control, XA21-virulent strain PXO99ΔraxST, grows to significantly higher numbers than both PXO99 and PXO99Δax21 at 8 and 12 days after inoculation (dai). On TP309 (right panel) the PXO99ΔraxST shows lower cell titer only at the 12 dai point. Each time point represents an average of 6 leaves ± SE. Statistical analysis was done for each time point using the Tukey-Kramer HSD test. Asterisk represents significant difference at p < 0.05. [file peerj-02-242-s006.png]
